# Optimization of textural characteristics of restructured pimiento strips by response surface methodology

**DOI:** 10.1002/fsn3.984

**Published:** 2019-03-28

**Authors:** Seyyed Mohammad Reza Mousavi, Ali Rafe, Samira Yeganehzad

**Affiliations:** ^1^ Department of Food Processing Research Institute of Food Science and Technology (RIFST) Mashhad Iran

**Keywords:** pimiento strip, responses surface methodology, restructured products, tensile, texture

## Abstract

In this study, the effect of guar gum (0.5%–1% *w*/*w*), sodium alginate (1%–2% *w*/*w*), and calcium chloride (2%–8% *w*/*w*) on textural properties of restructuring pimiento strips (RPS) was investigated. The gums were added to the pimiento strip formula, and different quality attributes including rupture force, energy to fracture, hardness, adhesiveness, cohesiveness, springiness, and chewiness were determined. Based on the textural properties of RPS, it was optimized by response surface methodology. All the textural properties of RPS were found to be significantly affected by alteration in guar gum, sodium alginate, and calcium chloride. The regression models for product’s response like rupture force and energy to fracture were highly significant. Results showed that restructured pimiento strip formula containing guar gum 1%* w*/*w* along with sodium alginate 2% *w*/*w* and 8% calcium chloride improved the textural and tensile properties. According to the RSM results on the textural properties of RPS, it is feasible to achieve the high elasticity and rigidity of pimiento strips as well as obtain the ability to tolerate thermal and mechanical stresses with appreciable textural integrity during processing such as pasteurization that would be investigated in another work.

## 1 INTRODUCTION

In the recent years, restructuring process is a key aspect of the food science, including complex combination of raw materials, components, and number of texturizing and construction processes (Laaman, 2011; Raharjo et al., 1995). In restructured products, a key material which is usually a natural raw material (e.g., vegetable, meat) is re‐formulated and the product is further processed. The aim of developing these products is to take natural food apart and then rebuilds them again in order to achieve better properties while the appearance, texture, and flavor are maintained (Laaman, 2011). In other words, by the process of restructuring, food can be produced with novel characteristics that did not exist in the main food (Laaman, 2011). For example, it can be pointed out that restructured food is able to tolerate thermal processes such as pasteurization, sterilization, while the raw material of the product does not have such properties. Moreover, the restructured food can be designed in such a way to stable at different conditions of preservation or storage. As a case, reconstructed pimiento strip can be pasteurized in jars and stable in the acidic calcium solution and keeps its texture characteristics up to time required for consumption (Laaman, 2011).

In preparation of stuffed olive pimiento, the process requires the elimination of stones and the creation of a cavity inside olive, and then, the cavity is filled with a piece of restructuring pimiento strip. The strips should be designed in such a way to have adequate strength, and they can easily cut without tearing. Therefore, they must be strong enough to protect from mechanical and thermal damages such as tearing, and also, they should withstand the pasteurization process and keeping the quality of product appearance with little syneresis and shrinkage (Laaman, 2011; Rees, 1987).

Nowadays, textural properties of gelled food are important challenges of consumer reception. Although there are varying procedures developed to evaluate the textural properties, the most generally used method is texture profile analysis (TPA)(Bourne, 2002; Kilcast, 2004). Among TPA tests, tensile stress can proffer noteworthy and complete data compared to those obtained using traditional instrumental texture methods (Herrero et al., 2007). Thus, tensile test can be used to study the mechanical properties of alginate restructured fibrous food (Harper, Barbut, Lim, & Marcone, 2013). Tensile properties, such as breaking strength and energy to fracture, are important parameters of the quality in restructured food due to the increasing tendency of the marketing restructured products (Herrero et al., 2008). The main challenge of producing restructured strips of “pimiento ribbon or strip” is to break them easily during handling, processing, and thermal treatment. If the breaking strength or the energy to fracture were less than that of the superficial adhesion force between the product and the surface of the processing equipment, another problem may arise regarding which products could break and led to stop the processing line of the restructured products. For example, when the force of adhesion of the product to packaging material or to the product is more than the breaking strength or the energy to fracture, the products undergo distortion, disfigurement, and breakage, producing adverse reactions in consumers (Herrero et al., 2008).

Response surface methodology (RSM) is a compilation of statistical and mathematical techniques that are suitable for designing, modeling, and optimizing processes (Myers, Montgomery, Vining, Borror, & Kowalski, 2004). The field of response surface methodology consists of the experimental strategy for exploring the space of the process or independent variables, empirical statistical modeling to develop an appropriate relationship between the response and the process variables, and optimization methods for finding the values of the process variables that produce desirable values of the response (Carley, Kamneva, & Reminga, 2004). RSM has been widely applied in several fields, particularly in food science (Sivakumar, Manohar, & Divakar, 2007) and environmental engineering (Trinh & Kang, 2010).

To the best of my knowledge, no research has been performed on the mechanical and textural properties of restructuring pimiento strips consist of alginate–guar gum. Therefore, the objectives of this research were to investigate the effects of three process variables, sodium alginate, guar gum, and calcium chloride concentrations on the textural properties of pimiento strip; and determine the optimum levels of sodium alginate, guar gum, and calcium chloride for the texturized formula by using RSM.

## 2 MATERIALS AND METHODS

### 2.1 Materials

Pimiento (*Capsicum Annuum* L.) was procured from a local supermarket of Mashhad, Iran. Pimiento puree was achieved by using a home juicer (MJ‐W176P; Panasonic, Japan). The chemical characteristics of pimiento pulp including pH, acidity, and total dissolved solids (TDS) were determined according to the Cepeda, García, Renobales, and Costell, (2000) (Table 1). Sodium alginate from brown algae (mannuronic/guluronic ratio of ~1.56, polymerization degree of 400–600, molecular weight of 80,000–120,000, and medium viscosity) was supplied from Sigma‐Aldrich (Lot No: BCCG6792V; St. Louis, MO, USA). Guar gum was purchased from Sigma‐Aldrich (Lot No: 063M8129V). Calcium chloride, sodium chloride, and citric acid were provided from Merck (Lot No: K46819478542, K40124804919, and K6092221531). Lactic acid was purchased from Fluka (Lot No: STBB3401V). Other reagents used in this work were of analytical grade.

**Table 1:** Physicochemical analysis of experimental pimiento pulp

Property	Pimiento pulp
pH	4.91 ± 0.20
Total acidity (g citric acid/100 ml)	0.19 ± 0.05
Total dissolved solids (TDS) (%)	4.2 ± 0.2

### 2.2 Pimiento strip preparation

The pimiento strips were prepared according to our previous work (Mousavi, Rafe, & Yeganehzad, 2019). In brief, pimiento puree was thoroughly mixed with deionized water, potassium sorbate (0.1 g), sodium alginate, and guar for 5 min at 21–48 *g* over a juicer blender (SHARP ‐ COUNTERTOP BLENDER SBTI172G) in order to achieve no lumps and the mixture became homogenous. Then, the mixture was rest for 30 min to remove the entrapped air bubbles. The product, viscous pimiento paste, was extruded by a head cut laboratory syringe (Mousavi et al., 2019) under gravity into the setting bath containing calcium chloride at concentrations 2.0%, 5.0%, and 8.0% *w*/*w* to develop a wide continuous sheet with 2 mm thickness. The setting sheet moved along the bath propelled by more paste was extruded. At the end of setting bath, the strip was left for 20 min to complete the gelation. In order to obtain the proper gel strength, the strips were stored in the aging bath containing NaCl 8% *w*/*w* and CaCl_2_ 2% *w*/*w* in the presence of citric acid 1% *w*/*w* at room temperature to complete evenly the gelation for 1 week. Essentially, this is a rearrangement of the gel network (Skjåk‐Braek, Grasdalen, & Smidsrød, 1989). The effect of sodium alginate, guar gum, and calcium chloride levels on the tensile and textural properties of pimiento strips was investigated (Table 2).

**Table 2:** Independent variables and natural levels used for central composite rotatable design

Independent variables, %	Independent variables				
	−α	Low	Medium	High	+α
	1.68	−1	0	1	+1.68
Sodium alginate	0.84	1	1.5	2	2.16
Guar gum	0.42	0.5	0.75	1	1.08
Calcium chloride	1.05	2	5	8	8.95

### 2.3 Textural properties

#### 2.3.1 Tensile test

Tensile test was performed using Texture Analyser (TA.XT Plus; Stable Micro Systems Ltd., Godalming, UK) equipped with a 100 N digital force gauge. Rectangular strips (20 × 50mm) of each formula were cut and clamped between tensile grips. The initial distance between grips was 30 mm, and the crosshead speed was 0.5 mm/s (Karki, 2011). From force–time curves, rupture force (RF) and energy to fracture (EF) were determined. RF was taken as the maximum force peak height (N) required to break the sample, and EF was calculated as the area under the deformation curve (Herrero et al., 2008).

#### 2.3.2 Texture profile analysis and fractural properties of gels

Pimiento strips were taken out of the aging bath and cut into uniform square shape approximately 400 mm^2^. Uniaxial single compression tests were carried out at ambient temperature (23°C) using a Texture Analyser with a 50 kg load cell (TA.XT Plus; Stable Micro Systems Ltd.), which was attached to the computer software Stable Micro Systems (version 6,1,14,0)(Huang & Hsieh, 2005). In TPA process, parameters are set as follows: pretest speed, 2.0 mm/s; test speed, 0.5 mm/s; post‐test speed, 10.0 mm/s; and distance, 50% of original gel height. Two continuous compressions were performed for each pimiento strip, and TPA texture attributes (hardness, adhesiveness, cohesiveness, springiness, and chewiness) were extracted from the time–force curve (Huang & Hsieh, 2005).

### 2.4 Experimental designs and statistical analysis

Response surface methodology (RSM) was adopted in the experimental design. The central composite rotatable design for three independent variables of sodium alginate, guar gum, and calcium chloride concentration was selected. Each independent variable was used according to the literature study. The complete design is given in Table 3. Modified quadratic model was used for three‐factor design which is given in Equation (1):

(1) $$Y=\beta_{0}+\beta_{1}+\beta_{2}x_{2}+\beta_{3}+\beta_{11}x_{1}^{2}+\beta_{22}x_{2}^{2}+\beta_{33}x_{3}^{2}+\beta_{12}x_{1}.x_{2}+\beta_{13}x_{1}.x_{3}+\varepsilon$$

**Table 3:** Central composite rotatable design with process variables and experimental results of pimiento strip

Run no	Alginate (%)	Guar (%)	Calcium (%)	Hardness (g)	Adhesiveness (g/mm)	Cohesiveness (–)	Springiness (mm)	Chewiness (g/mm)	Rupture force (N)	Breaking strength (N/cm^2^)	Energy to fracture (g/s)
1	1.50	0.75	5.00	2,132.139	22.669	0.822	2.383	4,176.57	404.324	1,010.810	9,995.611
2	1.50	1.08	5.00	2,892.321	40.373	0.696	2.432	4,894.47	575.516	1,438.790	17,162.350
3	1.00	1.00	8.00	2,393.795	25.285	0.749	2.173	3,896.74	262.736	656.840	7,636.780
4	2.00	0.50	8.00	1,680.662	20.172	0.890	2.782	4,161.3	731.68	1,829.200	23,970.680
5	2.00	1.00	8.00	2,846.422	27.944	0.823	2.288	4,015.62	701.077	1,752.693	23,794.900
6	1.00	1.00	2.00	1,771.949	24.229	0.771	2.382	3,253.62	221.714	554.285	5,440.307
7	0.84	0.75	5.00	2,050.724	37.732	0.750	2.074	3,190.01	217.556	543.890	5,915.699
8	1.00	0.50	2.00	1,889.891	27.469	0.768	1.945	2,824.51	189.833	474.582	4,661.603
9	1.50	0.75	5.00	2,194.054	18.204	0.812	2.345	4,178.92	494.275	1,235.688	13,681.500
10	2.00	1.00	2.00	1,385.604	19.643	0.888	2.898	3,563.92	790.216	1,975.540	27,023.570
11	2.16	0.75	5.00	2,319.301	36.483	0.836	2.736	5,307.26	838.542	2,096.355	26,607.830
12	1.50	0.75	5.00	2,132.337	23.348	0.810	2.403	4,150.35	480.547	1,201.368	12,832.490
13	1.00	0.50	8.00	2,179.362	28.618	0.757	2.082	3,436.23	272.573	681.433	7,686.823
14	2.00	0.50	2.00	2,377.581	11.159	0.776	2.722	5,019.27	820.227	2,050.568	28,593.050
15	1.50	0.75	8.95	2,956.686	29.954	0.776	2.007	4,601.96	459.677	1,149.193	13,278.320
16	1.50	0.75	5.00	2,110.250	19.850	0.836	2.339	4,128.38	480.547	1,201.368	12,832.490
17	1.50	0.75	1.05	1,985.615	29.546	0.817	2.537	4,113.13	328.112	820.280	8,538.265
18	1.50	0.75	5.00	2,201.256	20.230	0.752	2.331	3,858.11	438.272	1,095.680	11,898.920
19	1.50	0.42	5.00	2,748.793	29.092	0.779	2.036	4,361.44	565.877	1,414.693	19,115.210
20	1.50	0.75	5.00	2,156.250	18.950	0.767	2.451	4,055.51	438.272	1,095.680	11,898.920

where *Y* is the predicted response, *β*_0_, *β*_1_, *β*_2_, *β*_3_, *β*_11_, *β*_22_, *β*_33_, *β*_12_, and *β*_13_ are the coefficients for linear, quadratic, and interaction terms, respectively, and *ε* is the residual error. *x*_1_, *x*_2_, and *x*_3_ are the real values of independent variables, that is, sodium alginate, guar gum, and calcium chloride concentrations. In the current work, hardness (H), adhesiveness (A), cohesiveness (Co), springiness (S), chewiness (C), rupture force (RF), and energy to fracture (EF) were determined. The statistical analysis was carried out using Design‐Expert 10 (version 10 by STAT‐EASE inc., USA). To check the adequacy of regression model, *R* ^2^, adjusted *R* ^2^, adequate precision, and Fisher’s *F* test were used (Montgomery, 2001). The confidence level of the experiments was selected at 95%.

## 3 RESULTS AND DISCUSSION

Variation of response (hardness, adhesiveness cohesiveness, springiness, chewiness, rupture force, and energy to fracture) of pimiento strips with independent variables (sodium alginate, guar gum, and calcium chloride concentration) was analyzed and shown in Table 3. The result of regression analysis is shown in Table 4.

**Table 4:** Estimated regression coefficient^*^

Parameters	H (g)	A (g/mm)	Co (–)	S (mm)	C (g/mm)	RF(N)	EF (g/s)
*β*0	+1,578.75245	+89.59969	+0.63490	+0.2292	+6,121.31128	+168.67128	+12,383.75
Alginate% (A)	+2,266.24182	−61.27166	−0.12746	+0.6035	−117.97903	+251.21930	+9,175.52
Guar% (B)	−1,914.88280	−57.93028	+0.56332	+2.37078	−23,984.28414	−1,365.34274	−74,643.02348
Calcium% (C)	−225.39545	+0.46752	−1.79528E−003	+0.14838	+251.01690	+91.77397	+2,979.62764
*A* ^2^	−731.64471	+10.97089	+0.044094	+0.24995	−885.56426	+127.56170	+7,897.14614
*B* ^2^	—	+21.94425	−0.33754	—	+18,477.86623	+904.20753	+48,927.71104
*C* ^2^	—	−0.16724	+1.40521E−003	—	—	−5.06263	−124.06022
AB	—	+22.82900	+0.050856	−0.84600	+16,025.46539	—	—
AC	—	+1.25908	+6.90616E−003	−0.039833	−138.42518	−25.12067	−1,089.39442
BC	+415.01867	−0.13417	−0.031578	−0.16933	—	—	—
AB^2^	—	—	—	—	−12,344.07726	—	—
*R* ^2^	0.5776	0.3315	0.6348	0.8990	0.7344	0.9778	0.9719
Lack of fit (*p* value)	109.59	30.82	1.94	8.60	20.42	1.45	2.10

Note

A: adhesiveness; C: chewiness; Co: cohesiveness; EF: energy to fracture; H: hardness; RF: rupture force; S: springiness.

### 3.1 Hardness

Hardness of strips was determined by measuring the force in gram required to break the product, and it varied from 1,385 to 2,956 g. The hardness of the product is a sensory perception of the humans and is correlated with development. The effect of sodium alginate, guar gum, and calcium chloride concentrations on the hardness is represented in Table 3. Regression analysis showed that hardness of strips was significantly increased by the amount of calcium ion, although no significant effect was found by the guar gum and sodium alginate (Table 4). Second‐order nonlinear regression model showed a good relationship. The lack of fit of the model was achieved at the 0.01% level and estimated coefficient of variation was 57.76%, which indicates that a quadratic model can be used to express the relation. Increasing in hardness can be related to the more cross‐linking of the alginate by calcium, which improves the gel strength. Similar results were also reported in our previous work (Mousavi et al., 2019).

**Figure 1:**
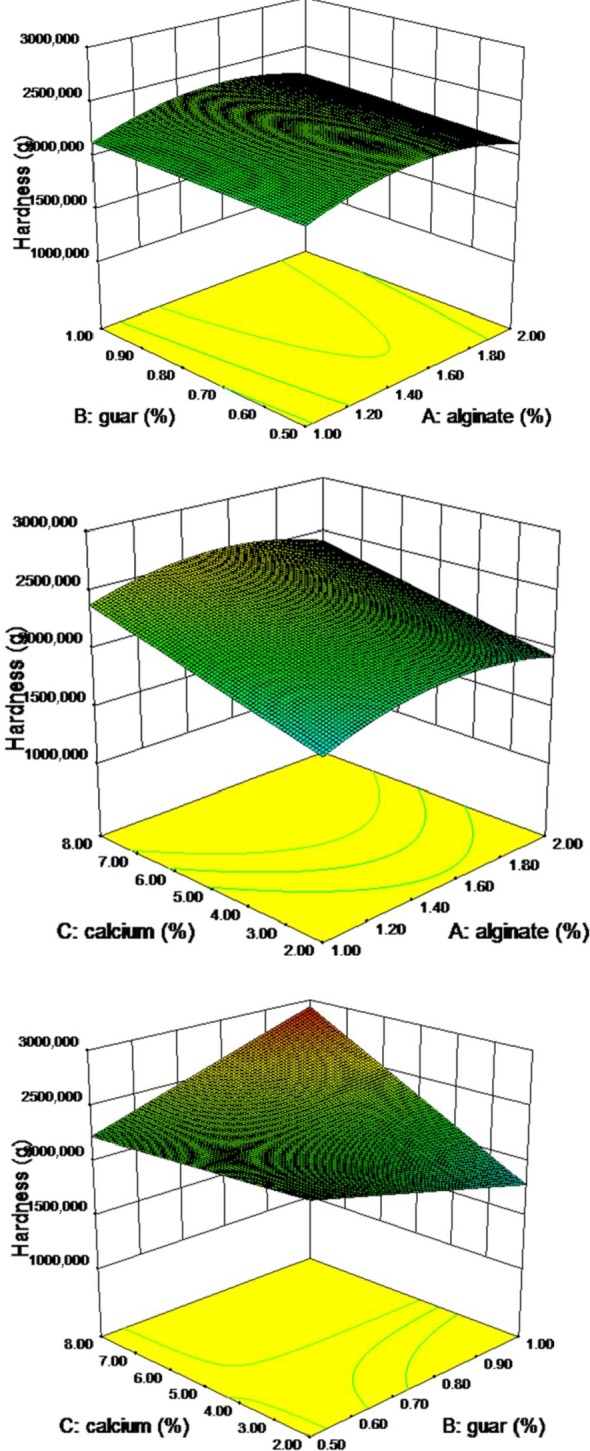
Response surface plot for hardness as a function of calcium, guar, and sodium alginate levels

The results showed that the model is significant in the *F* value of 3.83. The effect of calcium ion and guar gum interactions with calcium was significant on the hardness of pimiento gel (Figure 1). In other words, this interaction showed a positive effect on the hardness of the gel. The coefficient of variation was 13.88% and model accuracy was more than 7.165 which is higher than 4 are desirable, and both coefficeints showed the model is suitable. The hardness prediction model for the pimiento gel is recommended as the following equation (Equation (2)):

(2) H=1578.75+2266.24A−1914.88B−225.39C+415.01BC−731.64A

### 3.2 Adhesiveness

**Figure 2:**
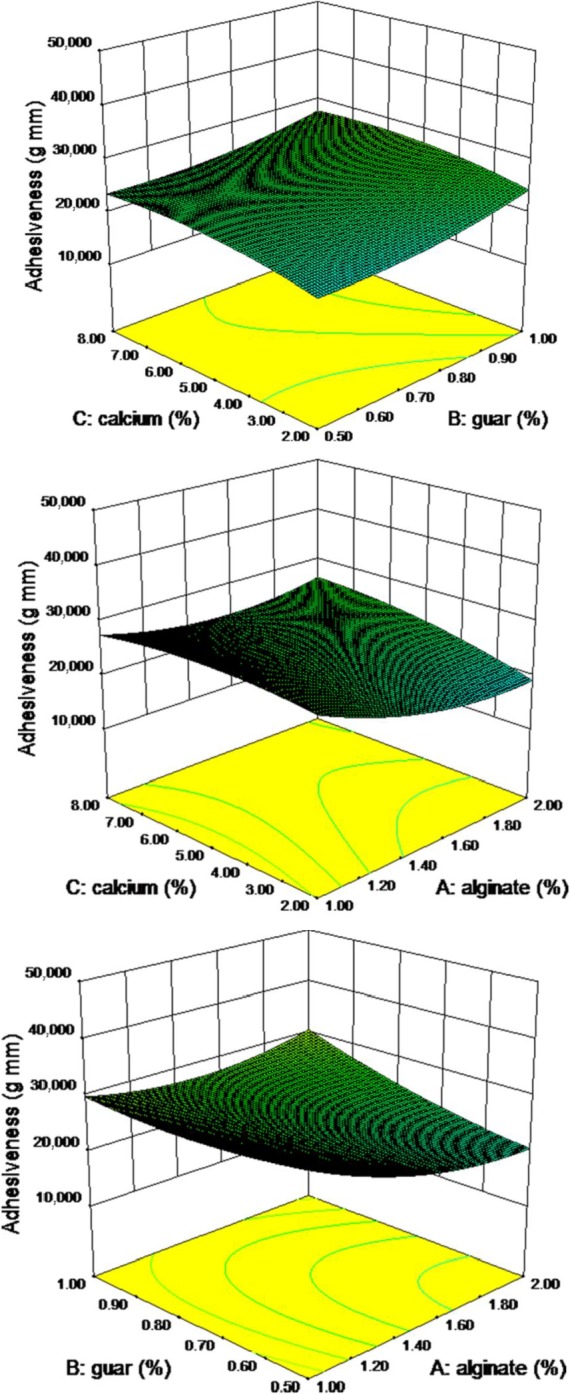
Response surface plot for adhesiveness as a function of calcium, guar, and sodium alginate levels

Adhesiveness of strip gels was determined by measuring work necessary to overcome the attractive forces between the surface of the pimiento strips and the surface of the plate of Texture Analyzer which comes in contact with it, and it varied from 11 to 40 g/mm. The effect of sodium alginate, guar gum, and calcium chloride concentrations on the adhesiveness is represented in Table 3. All adhesiveness values were negative, and the least adhesiveness was obtained at the lowest concentration of calcium. Thus, less work is required to pull the compressing cylinder probe away from the gel sample at 2% level of calcium chloride. Nonlinear polynomial curve relationship between the variables and the response was achieved, and the analysis of variance showed a significant effect of variables on the adhesiveness of the pimiento gel at *p* ≤ 0.05 (Table 4). However, adhesion was decreased by increasing alginate levels and increased by increasing guar and calcium chloride (Figure 2). The analysis of variance of adhesiveness of the pimiento strip is presented in Table 4. Second‐order nonlinear regression model does not show a desirable relationship. The lack of fit of the model is not achieved at 0.01% level, which can be due to error, and the coefficient of variance is 33%. The results showed that the model is not significant in the *F* value of 0.55. A negative “Pred *R* ^2^” implies that the overall mean may be a better predictor of this response than the current model. The coefficient of variation was 333%, and model accuracy was 3. The adhesiveness prediction model for the pimiento gel is recommended as the following Equation (3):

(3) Adhesiveness=89.59−61.27A−57.93B+.42C+22.82AB+1.259AC−.13BC+10.97A2+21.94B2−.16C2

### 3.3 Cohesiveness

**Figure 3:**
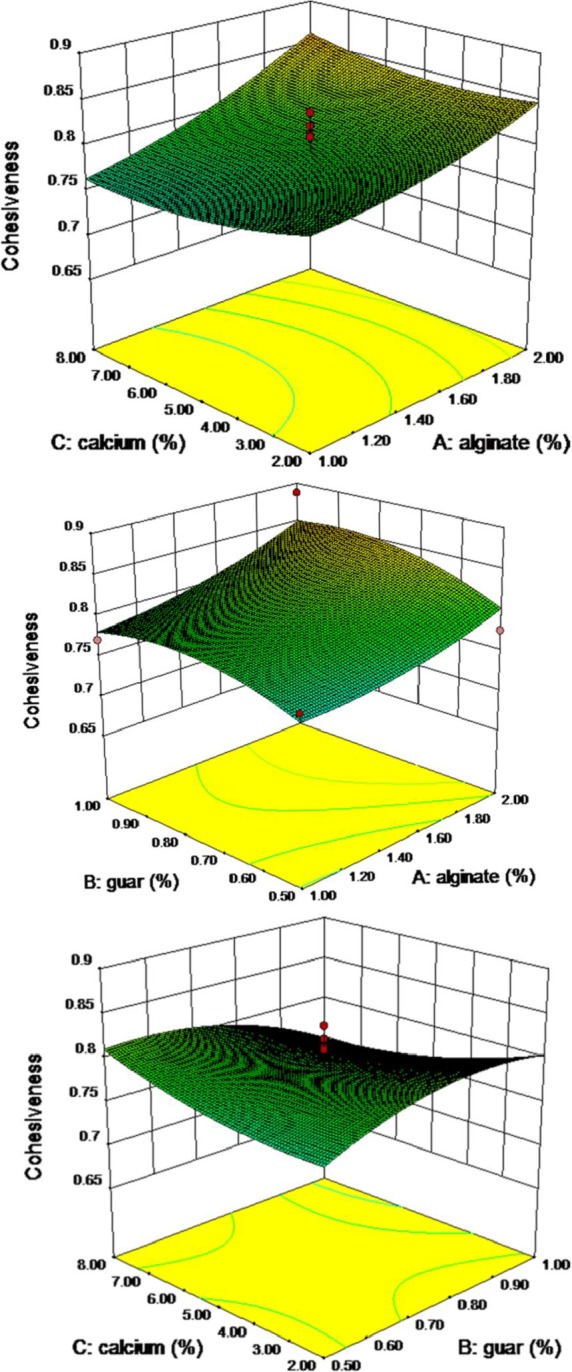
Response surface plot for cohesiveness as a function of calcium, guar, and sodium alginate levels

Cohesiveness can be explained as a measurement of how well the structure of a product withstands compression, the work needed to break internal bonds. It can be defined as extent to which pimiento strip can be deformed before it ruptures, and it varied from 0.70 to 0.89. It is anticipated that the more the structure is deformed, the more the internal bonds might be broken. The effect of sodium alginate, guar gum, and calcium chloride concentrations on the cohesiveness is presented in Table 3. Nonlinear polynomial curve showed relationship between the variables and the response, and the analysis of variance showed a significant effect of variables on the cohesiveness of the pimiento gel at *p* ≤ 0.05 (Table 4). Increasing the concentration of alginate has a positive effect on gel cohesiveness. However, guar gum did not have a significant effect, and to some extent, it was increased and then decreased, which had the opposite effect response for calcium chloride. Thus, it was slightly reduced then ineffective and it was finally increased (Figure 3). Second‐order nonlinear regression model showed a good relationship. The lack of fit of the model was achieved at 0.01% level. The results showed that the model is not significant in the *F* value of 1.93. A negative “Pred *R* ^2^” implies that the overall mean may be a better predictor of this response than the current model. The coefficient of variation was 5.04% and model accuracy was more than five which is higher than four and it is desirable, which shows that both models are suitable. The cohesiveness prediction model for the pimiento gel is recommended as the following Equation (4):

(4) Cohesiveness=0.63−0.127A+0.56B−0.00179C+0.05AB+6.9AC−0.03BC+0.04A2−0.33B2+1.4C2

### 3.4 Springiness

Springiness is rate at which a deformed pimiento strip gel goes back to its undeformed condition after the deforming force is removed, and it varied from 1.94 to 2.90 (Table 3). As it can be found from Table 3, springiness was initially decreased at 5% of calcium chloride and then increased at 8%. Moreover, the springiness decreased with increasing alginate levels in the calcium chloride–alginate gels (Wang, 2015). The initial reduction in springiness may be related to the increasing of the gel hardness. As the alginate and calcium levels increased, a stronger gel structure formed and provided more resistance to compression, which resulted in less deformation and therefore, after compression, the gels would recover less. However, increase in springiness was associated with gel hardness at 8% calcium level. Springiness of pimiento strip was also much lower than the carrot pulp restructured alginate gels (Manjunatha & Das Gupta, 2006), which can be attributed to the effect of guar gum in the alginate gel systems.

**Figure 4:**
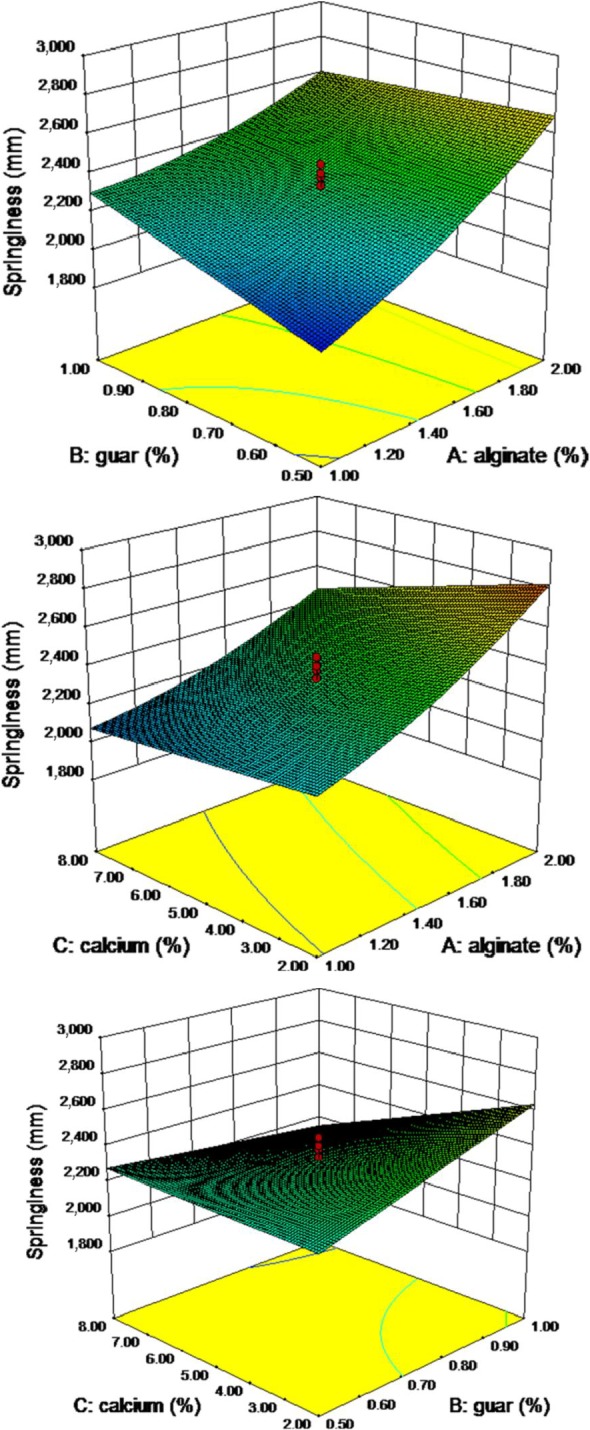
Response surface plot for springiness as a function of calcium, guar, and sodium alginate levels

Second‐order nonlinear regression model showed a good relationship, and the resulted analysis of variance showed a significant effect of variables on the springiness of the pimiento gel at *p* ≤ 0.05 (Table 4). Increase in the concentration of alginate and guar gum has a positive effect on the gel springiness. However, guar’s effect is more moderate than that of alginate. Gel springiness decreases with increasing amount of calcium ion, but the binary interaction of alginate and guar, as well as guar and calcium chloride, has a significant effect on gel resistance (Figure 4). Second‐order nonlinear regression model showed a desirable relationship. The lack of fit of the model is not achieved at 0.01% level. The results showed that the model is significant in the *F* value of 15.26. Calcium ions and alginate were significant parameters of the model. The “Pred *R* ^2^” of 0.55 is not as close to the “Adj *R* ^2^” of 0.84 as one might normally expect; that is, the difference is more than 0.2. This may be representative of a large block effect. The coefficient of variation was 4.57%, and model accuracy was more than 14 which is higher than four are desirable, which both show that model is suitable. The springiness prediction model for the pimiento gel is recommended as the following Equation (5):

(5) Springiness=0.229+0.603A+2.37B+0.148C−0.84AB−0.039AC−0.169BC+0.24A2

### 3.5 Chewiness

**Figure 5:**
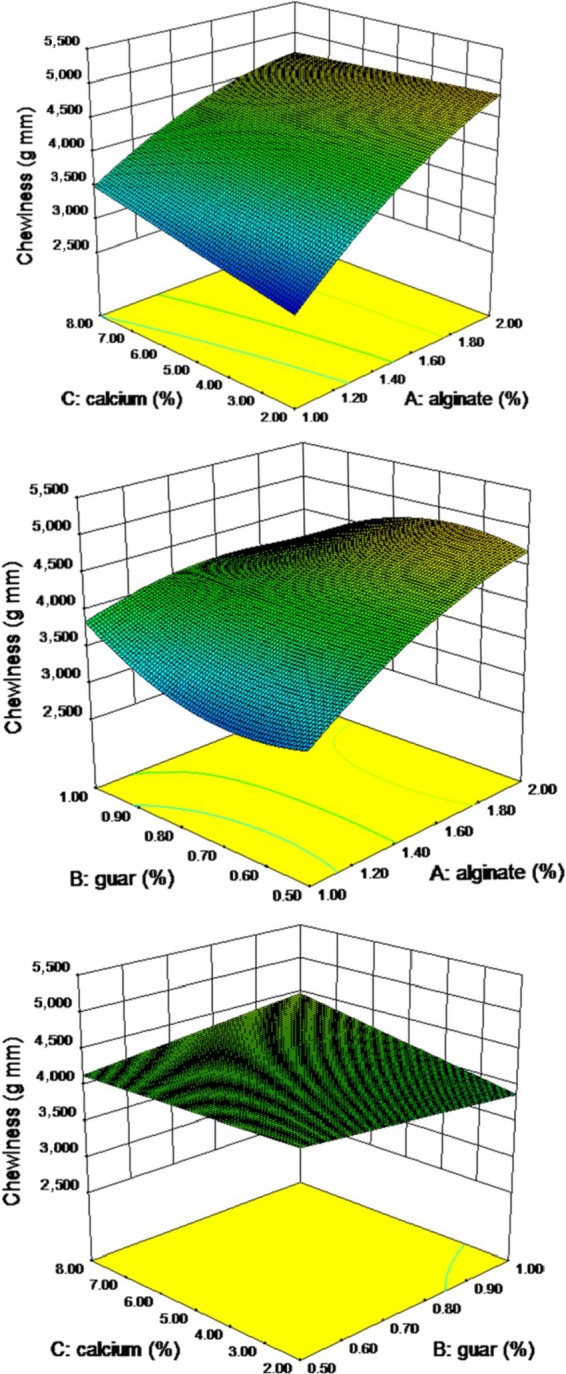
Response surface plot for chewiness as a function of calcium, guar, and sodium alginate concentrations

Chewiness is defined as energy required masticating pimiento strip gel to a state ready for swallowing: a product of hardness, cohesiveness, and springiness, and it varied from 1.94 to 2.90 (Table 3). Second‐order nonlinear regression model showed a good relationship, and the resulted analysis of variance showed a significant effect of alginate on the chewiness of the pimiento gel at *p* ≤ 0.05. However, calcium’s effect is more moderate than alginate, and the guar gum has no significant effect on the chewiness. At the same time, the interaction between alginate and guar had a significant effect (Figure 5). Second‐order nonlinear regression model showed a good relationship. The lack of fit of the model was not achieved at 0.01% level. The results showed that the model is significant in the *F* value of 3.8. The coefficient of variation was 10.31% and model accuracy was more than 7.5 which was higher than 4 and showed the model is suitable (Table 4). The chewiness prediction model for the pimiento gel is recommended as the following Equation (6):

(6) Chewingness=6121−117A−23984B+251C+16025AB−138AC−885A2+18477B2−12344AB2

### 3.6 Rupture force

**Figure 6:**
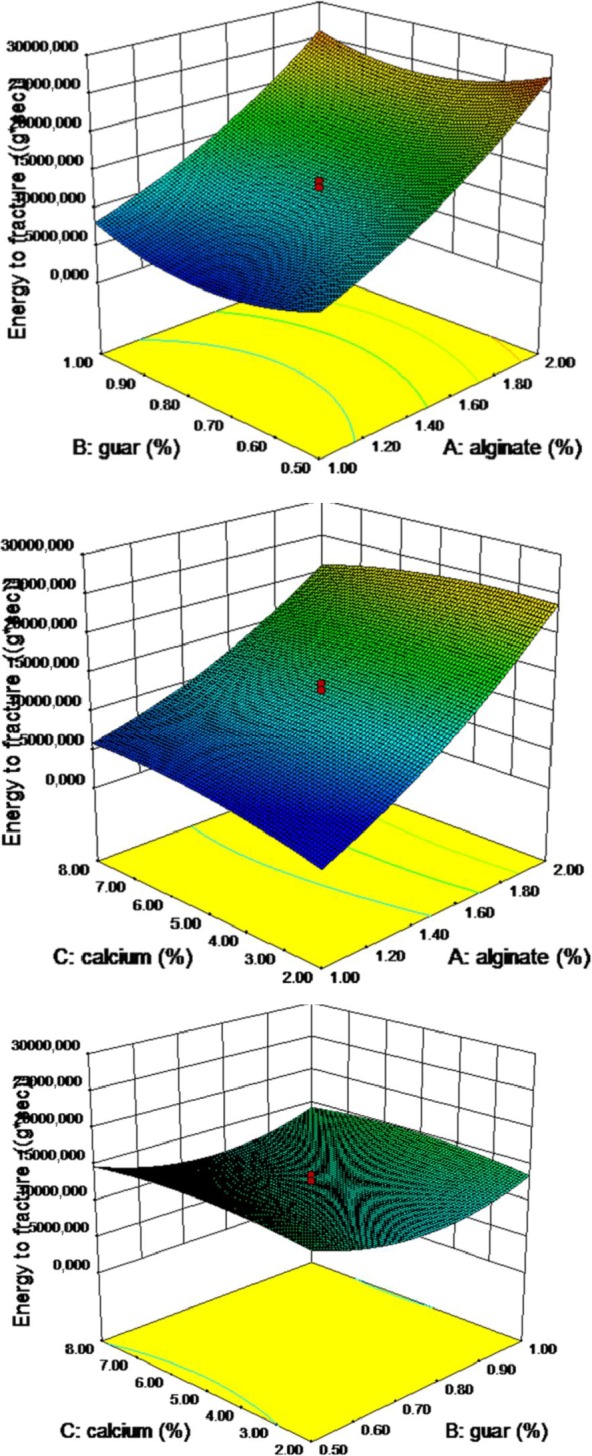
Response surface plot for energy to fracture as a function of calcium, guar, and sodium alginate levels

Rupture force is maximum peak height resisted by pimiento gel, and it varied from 189 to 838 (N) (Table 3). Second‐order nonlinear regression model showed a good relationship, and the resulted analysis of variance showed a significant effect of variable on the rupture force of the pimiento gel at *p* ≤ 0.05. Increasing the concentration of alginate resulted in increased fracture force (Figure 6). In addition, guar and calcium chloride concentration did not influence on the rupture force. However, the binary interaction of alginate and calcium chloride and C^2^ resulted in a reduction in the gelling strength of the gel. However, A^2^ and B^2^ resulted in increasing the gel fracture. The analysis of variance of rupture force of the pimiento strip is presented in Table 4. Second‐order nonlinear regression model showed a good relationship. The lack of fit of the model was achieved at 0.01% level and estimated coefficient of variation was 7.99%, which indicates that a quadratic model can be used to express the relation. The results showed that the model is significant in the *F* value of 7.99, and model accuracy was more than 27, which showed the model was suitable (Table 4). Rupture force prediction model for the pimiento gel is recommended as the following Equation (7):

(7) Ruptureforce=168.67+241.21A−1365B+91.77C−25.12AC+127.56A2+904B2−5.06C2

### 3.7 Energy to fracture

**Figure 7:**
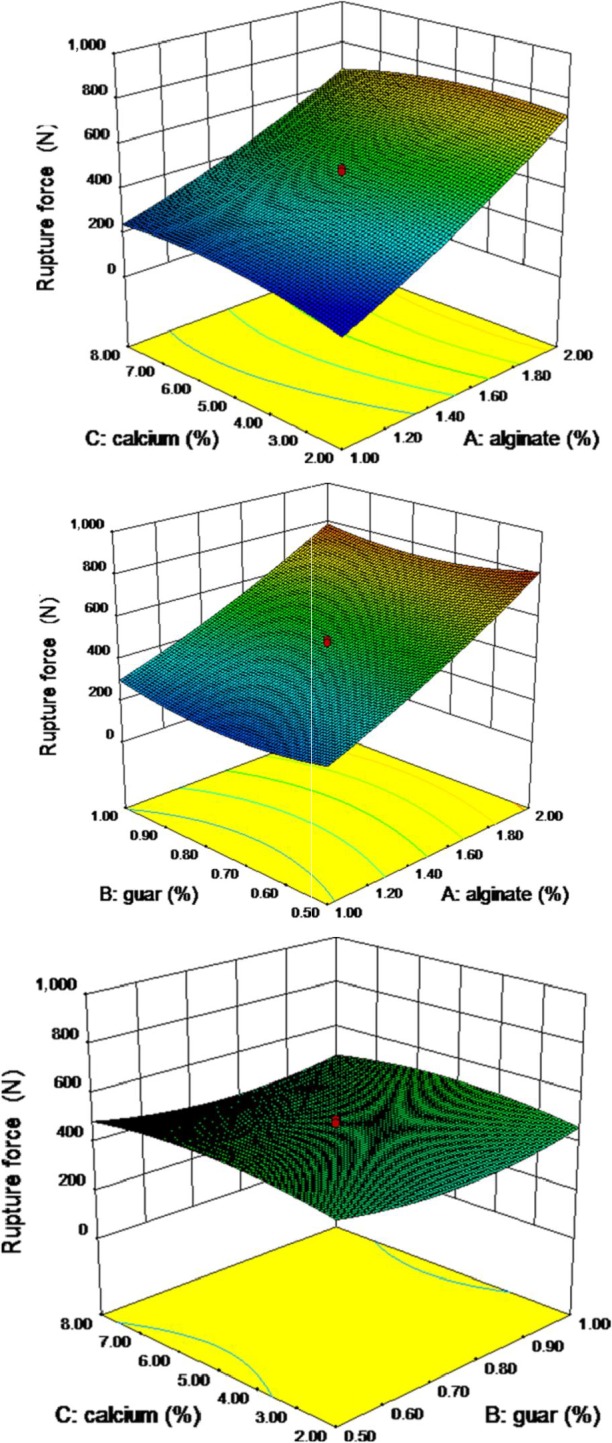
Response surface plot for rupture force as a function of calcium, guar, and sodium alginate

Energy to fracture of pimiento ribbon is defined as area under the deformation “force–time” curve, and it varied from 4,461 to 28,593 (g/s) (Table 3). Second‐order nonlinear regression model showed a good relationship, and the resulted analysis of variance showed a significant effect of variable on the energy to fracture of the pimiento gel at *p* ≤ 0.01 (Table 4). Increasing the concentration of alginate resulted in increased energy to fracture (Figure 7). The lack of fit of the model was not achieved at 0.01% level and the model is significant in the *F* value of 59.34, which shows that model is suitable. Alginate–guar, alginate, A^2^, and B^2^ are significant parameters of predicted models. It should be noted that coefficient of variance is 11.11% and the model’s accuracy was more than 23, which both indicate the suitability of the model. Energy to fracture prediction model for the pimiento gel is recommended as the following Equation (8):

(8) Energytofracture=19289+106A−74643B+2979C−1089AC+7897A2+48927B2−124C2

## 4 CONCLUSION

Results showed that the gel strength of alginate–guar gum system increased considerably by adding more calcium ion. Indeed, calcium lactate–alginate gels exhibited higher hardness than calcium citrate–alginate gels at the same calcium chloride level. In comparison with other restructured alginate gels, the guar gum as a thickening agent had a significant effect on the reduction of hardness. In order to achieve the elastic pimiento strip without any mechanical disruption, the alginate–guar gum at ratio 2:1 was found suitable. Hardness of strips was significantly increased by the amount of calcium ion, and the effect of calcium ion and guar gum interactions with calcium was significant on the hardness of pimiento strip. However, no significant effect was found on the thickener and sodium alginate. Nonlinear polynomial curve relationship between the variables and the response was achieved, and the resulted analysis of variance showed a significant effect of variables on the textural properties of the pimiento gels. Overall, the results completely approved that calcium level and the presence of guar gum can improve the elasticity and rigidity of pimiento strips at high value of fruit pulp as well as endure thermal processing such as pasteurization.

## CONFLICT OF INTEREST

The authors declare that they have no conflict of interest.

## ETHICAL APPROVAL

The study does not involve any human or animal testing.
